# First evidence of *Candidatus* Neoehrlichia mikurensis in Hungary

**DOI:** 10.1186/1756-3305-6-267

**Published:** 2013-09-17

**Authors:** Sándor Hornok, Marina L Meli, Enikő Gönczi, Regina Hofmann-Lehmann

**Affiliations:** 1 Department of Parasitology and Zoology, Faculty of Veterinary Science, Szent István University, Budapest 1078, Hungary; 2 Clinical Laboratory, Vetsuisse Faculty, University of Zurich, Zurich 8057, Switzerland

**Keywords:** Tick-borne diseases, Zoonosis, Epidemiology

## Abstract

Altogether 2004 *Ixodes ricinus* ticks, from 37 places in Hungary, were analysed in pools with a recently developed multiplex real-time PCR for the presence of *Candidatus* Neoehrlichia mikurensis and for other representatives of the genus. *Ca*. Neoehrlichia mikurensis was identified in nine sampling sites, indicating three separated endemic regions along the borders of Hungary. In addition, results of samples from seven places (except for the western part of the country) were positive in the genus-specific (*Ca*. Neoehrlichia sp.) PCR, but were negative for *Ca*. Neoehrlichia mikurensis.

## Letter

*Candidatus* Neoehrlichia mikurensis is a Gram-negative, coccoid bacterium in the family Anaplasmataceae that infects endothelial cells of its host [1]. In Europe it is transmitted by *Ixodes ricinus*. Larvae and nymphs of this tick species frequently suck blood on rodents, which are the most important wild animal reservoirs of *Ca*. Neoehrlichia mikurensis [2,3]. Subsequently, if nymphs or adults of *I. ricinus* will suck blood on humans or domestic animals, these hosts may become infected. *Ca*. Neoehrlichia mikurensis was isolated several times from diseased humans, justifying its zoonotic nature [4]. Among domestic animals the susceptibility of dogs was demonstrated [5].

In Europe the history of *Ca*. Neoehrlichia mikurensis dates back only 15 years. First indications of a related (*Ehrlichia*-like) sequence became available in 1998 from The Netherlands [6], then later (2003-2006) from Italy [5,7]. More recent studies showed the presence of this agent in several Western and two Central European countries: Switzerland, Belgium, Germany, Sweden, Denmark, the Czech Republic and Slovakia [8]. Even in Western Europe some countries, like the UK, appear to be exempt of *Ca*. Neoehrlichia mikurensis [2]. However, relevant information is lacking from most parts of Central-Eastern Europe.

For this reason it was decided to screen large numbers of *I. ricinus* ticks for the presence of *Ca*. Neoehrlichia mikurensis with a recently developed, highly sensitive molecular method [8].

**Figure 1:**
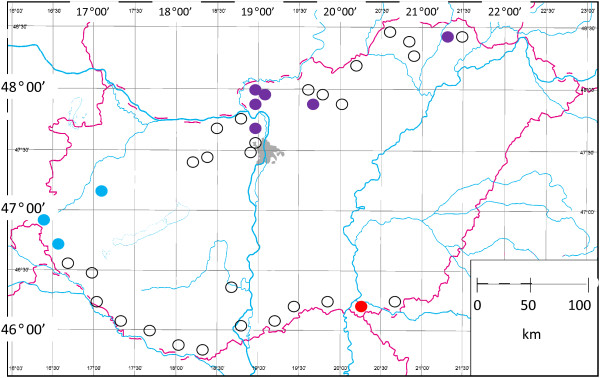
**Places of tick collection (circles) in Hungary.** Full circles indicate places with samples positive in the multiplex PCR according to the following colour codes: blue dots stand for places where only *Ca*. Neoehrlichia mikurensis was identified in its genus; red dot marks the place where *Ca*. Neoehrlichia mikurensis was not identified, but PCR positivity was detected on the genus level (*Ca*. Neoehrlichia sp.); purple dots indicate places, where both types of samples were collected (i.e., some of them positive to *Ca*. Neoehrlichia mikurensis and others positive to *Ca*. Neoehrlichia genus excluding known European sequences of *Ca*. Neoehrlichia mikurensis).

DNA samples of 2004 *I. ricinus* ticks were used in this study. These ticks were collected from the vegetation by the dragging-flagging method from March to July in 2007, at 37 locations in Hungary (Figure 1). A minimum of 40 ticks were collected from each location. The sampling focused on the southern and on the northern borders of Hungary, to monitor any epidemiological connections of tick-borne agents with neighbouring countries. Ticks were stored in 70% ethanol and pooled prior to DNA extraction according to places of collection.

DNA extraction from tick pools was done by the MagNA Pure LC total nucleic acid isolation kit (Roche Diagnostics, Rotkreuz, Switzerland). Amplifiable DNA contents of each tick pool were evaluated by a TaqMan real-time PCR for the 18S rRNA gene, as reported [9]. DNA samples were stored at -80°C until molecular analysis.

The presence of *Candidatus* Neoehrlichia mikurensis was investigated by a multiplex TaqMan real-time PCR [8]. In brief, the assay is based on the amplification of a 16S rRNA gene fragment shared by members of the family Anaplasmataceae, and on the simultaneous usage of three specific probes to indicate positivity on the family (Anaplasmataceae), the genus (*Ca*. Neoehrlichia) and species (*Ca.* Neoehrlichia mikurensis) level. Each PCR run included serial 10-fold dilutions of a synthetically produced 586 bp long gene (GeneArt® Strings™ DNA Fragments, Life Technologies, Paisley, UK) based on the sequence deposited in the GenBank [GQ501090], with known copy numbers for quantification.

*Ca*. Neoehrlichia mikurensis was detected in nine out of 37 places (Figure 1). Based on the geographical distribution of positive tick pools three endemic regions of *Ca*. Neoehrlichia mikurensis could be recognized in Hungary (Figure 1). Interestingly, ticks from seven places gave positive results in the *Ca*. Neoehrlichia genus PCR, but were negative in the *Ca*. Neoehrlichia mikurensis-specific assay. None of these places was located in Western Hungary, and in six out of seven *Ca*. Neoehrlichia mikurensis was also identified (Figure 1). The maximum copy number in these samples was 2.8 × 10^3^ (threshold cycle value 29), which was not enough for sequencing.

This is the first evidence of *Ca*. Neoehrlichia mikurensis in Hungary. Additionally, with the exception of an endemic focus in Slovakia [10], no information is available on the occurrence of this zoonotic pathogen in neighbouring countries, highlighting the importance of the present findings in connection with Austria and Slovenia. *Ca*. Neoehrlichia mikurensis has been reported in geographically close regions of various countries, like Belgium and The Netherlands [2], Sweden and Denmark [11,12], or Eastern-Switzerland and North Italy [7,8]. In this study three endemic regions of *Ca*. Neoehrlichia mikurensis were recognized along the borders of Hungary. In particular, the central endemic zone (48° N, 19° E) is situated within 60 km of the central Slovakian focus of *Ca*. Neoehrlichia mikurensis (Malá Lehota [10]), therefore they may be confluent (i.e., representing a single, trans-boundary endemic focus).

There were pools positive in the genus-specific PCR, but negative for *Ca*. Neoehrlichia mikurensis. This phenomenon was not observed previously when testing a similar number of tick specimens from Switzerland during the development of the method used in this study [8]. Taking into account the design of the genus- and species-specific probes, these results may indicate the existence of new sequevars of *Ca*. Neoehrlichia spp. [8], formerly not detected in Europe.

## Competing interests

The authors declare that they have no competing interests.

## Authors’ contributions

SH collected the ticks, extracted the DNA, and has written the manuscript. MM designed, and in part performed the molecular analyses. EG performed most of the molecular analyses. RHL initiated and supervised the study. All authors read and approved the final version of the manuscript.
